# Addressing the stability issue of perovskite solar cells for commercial applications

**DOI:** 10.1038/s41467-018-07255-1

**Published:** 2018-12-10

**Authors:** Lei Meng, Jingbi You, Yang Yang

**Affiliations:** 1 Department of Materials Science and Engineering, University of California, Los Angeles, 90095 CA USA; 2 Key Laboratory of Semiconductor Materials Science, Institute of Semiconductors, Chinese Academy of Sciences, 100083 Beijing, China; 3 College of Materials Science and Opto-electronic Technology, University of Chinese Academy of Sciences, 100049 Beijing, China

## Abstract

When translating photovoltaic technology from laboratory to commercial products, low cost, high power conversion efficiency, and high stability (long lifetime) are the three key metrics to consider in addition to other factors, such as low toxicity, low energy payback time, etc. As one of the most promising photovoltaic materials with high efficiency, today organic–inorganic metal halide perovskites draw tremendous attention from fundamental research, but their practical relevance still remains unclear owing to the notorious short device operation time. In this comment, we discuss the stability issue of perovskite photovoltaics and call for standardized protocols for device characterizations that could possibly match the silicon industrial standards.

## The golden triangle

Organic–inorganic metal halide perovskite solar cells (PSCs), usually represented by methylammonium lead triiodide (MAPbI_3_), have witnessed great achievement since the first demonstration of PSC in 2009. The certified power conversion efficiency (PCE) has risen from 14.1 to 23.3% within a few years, which is the fastest growing photovoltaic (PV) technology in history^1^.

**Fig. 1:**
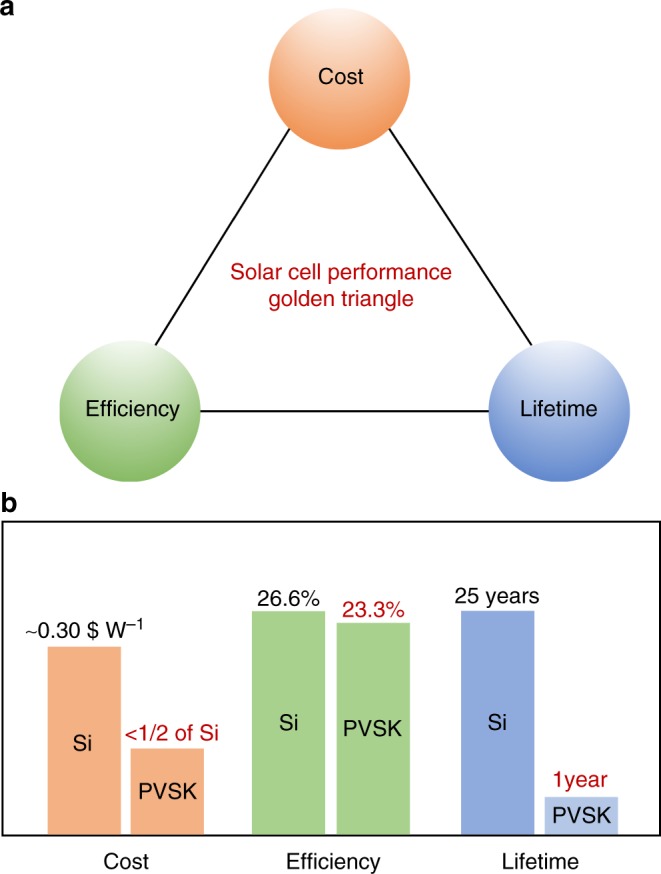
The comparison of perovskite and silicon solar cells. **a** Golden triangle of solar cells, cost, efficiency, and lifetime are considerd. **b** The comparison of perovskite and silicon solar cells based on golden triangle. Silicon solar cells have the champion efficiency of 26.6% (21% for the module) lifetime of more than 25 years and cost of around 0.3 $ W^−1^. In comparison, the perovskite solar cells achieve the champion efficiency of 23.3% (17% for the small module), the manufacture cost is around half of the silicon solar cells and the lifetime of only one year at present

Besides the efficiency, lifetime (or stability) and cost, i.e., the golden triangle, are considered to gauge the technical feasibility for commercialization of PV technologies (Fig. 1). More than 90% of the current market share of the commercialized PVs is taken by silicon PV because it delivers a package of decent module efficiency of 21%, long lifetime of more than 25 years and low cost of 0.3 $ W^−1^ that is reaching the grid parity. In comparison, perovskite single cells hold promise because of their efficiency reaching 23% and above and low manufacturing cost, which has been estimated to be able to reach the half of that of crystalline Si^2^. However, the stability of perovskite solar cells is quite problematic. So far, the longest lifetime reported for PSCs is about one year^3^, which is much shorter than 25 years as expected from commercialized PV technologies. It is thus clear that the short lifetime is the main obstacle hindering the commercialization of PSC PV^4^.

## Addressing the stability issue

The lifetime of PSCs is affected by many factors, which can be classified into two categories: extrinsic (environmental) and intrinsic factors. Environmental factors such as moisture and oxygen can be settled by encapsulation and the most critical issues are due to the intrinsic instability of the bulk perovskite material and the interface between the perovskite and the charge transport layers.

There are three main intrinsic factors leading to perovskite instability: hygroscopicity, thermal instability, and ion migration. The hygroscopicity is related to the environmental factors and can also be solved by encapsulation^5^. The thermal instability can be addressed by composition tuning to increase the decomposition energy or barrier, e.g., with FA cations^6^. Lastly, the issue of ion migration is currently treated by A site alkali doping^7^ and replacement^5,8^, multiple dimensional perovskites engineering (MDPs)^3,9,10^, and organic molecular additives^11^. In fact, the ion migration is almost unavoidable in all halide perovskites due to the high external field applied across the thin films during the J-V scan and the high ionic mobility, and the situation is worse at the defective sites, grain boundaries, and the interfaces. Nevertheless, we believe the ion migration could be impeded or even prevented by passivating the grain boundary^11^, higher sample quality (reducing the grain boundary), and most promisingly, increasing the ion migration barrier by engineering the packing density of the crystal lattice via ion substitution^12^.

Charge transport layers are in direct contact with the photoactive perovskite layer and should protect it from environmental factors such as moisture, heavy metal ions in the electrodes besides their charge transporting functions. Currently, the most commonly used hole transport layer Spiro-OMeTAD must be replaced due to its high hygrpscopicity, tendency to crystalize, and vulnerability to both moisture and heat. So far, robust metal oxide^13,14^, carbon^3,15^, and other inorganic materials^6^ have been shown as efficient methods to increase the device stability, but in the meantime, the PCE in these devices remains to be optimized.

**Fig. 2:**
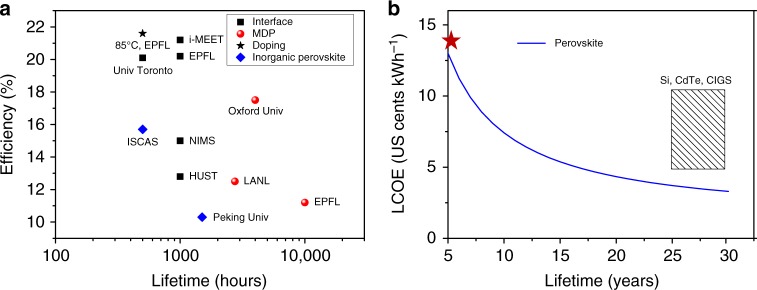
Stability and Levelized cost of energy (LCOE) of perovskite photovoltaic. **a** The state art of power conversion efficiency vs lifetime. Composition and interface engineering of perovskites have been adopted to improve the stability of perovskite solar cells. Interface: interface engineering including transport layer and electrode; MDP: multi-dimensional perovskite, Doping: inorganic cations doped in A site for ABX_3_ structure; Inorganic perovskite: CsPbI_3_ or CsPbBr_3_ or their mixture. The 10,000 h stability reported from EPFL is *T*_100_ stability, which means the device performance is not degraded during aging. EPFL: École Polytechnique Fédérale de Lausanne^3,6,7^, ISCAS: Institute of Semiconductors, Chinese Academy of Sciences^5^, Peking Univ^8^, LANL: Los Alamos National Laboratory^9^, Oxford Univ^10^. NIMS: National Institute Materials Science^13^ i-MEET: Institute of Materials for Electronics and Energy Technology^14^, HUST: Huazhong University of Science Technology^15^, Univ Toronto^16^. **b** LCOE of perovskite PV as a function of lifetime, and competitor PV technologies. The blue curve presents the estimated LCOE of perovskite solar cells as a function of their lifetime and the “red star” gives a rough estimation of the current situation. The LCOE curve is estimated using the “bottom-up”, assuming a module efficiency of 19%, major materials usage ratio of 80%. The module lifetime is set to be 20 years with 1% degradation per year. We simplify boundary conditions with respect to politics and finance, which are less relevant to the technical aspect. The discount rate (interest) is assumed to be 5% per year. No incentives, government subsidies, nor financing methods are considered

As a quick comparison, the resulted device efficiency and stability of above strategies are shown in Fig. 2a. The best lifetime obtained for perovskite solar cells is 10,000 h (around 1 year)^3^, but the PCE is only 12%. If we set an efficiency threshold of 20%, the best light-soaking stability is only 1000 h^6,14^. Our target can be set at efficiency of around 20% and a lifetime of 15 years to benchmark to Si PV (see the last section, The “real” cost of PSCs). Currently the device efficiency and stability are not simultaneously optimized, but there is no principle of physics prohibiting the achievement of both high efficiency and high stability in PSCs. We believe it is a matter of time to catch up with silicon given the tremendous momentum and the continuous input in the field of perovskite solar cells. The field has entered the phase where incremental and technical improvements should be appreciated. These incremental improvements will be accumulated to push the performance metrics to the limits and thus we believe all types of strategies are welcome.

## Unified stability tests and accelerated aging tests

While the research interest on the stability studies is growing rapidly, the published stability tests have been conducted in a wide range of non-standard conditions, making it impossible to compare the lifetime tests between different labs. Here we’d like to suggest “25 °C, encapsulated or inert gas protected device, maximum power point (MPP) tracing, AM 1.5 light soaking” as the standard test conditions to mimic the real scenario (device temperature could increase after light soaking, while for standard testing, a heat sink is applied to maintain constant temperature). Only a subset of standard test conditions is fulfilled in laboratory tests for perovskite solar cells^6,7,16^. For example, stability under certain relative humidity or ambient condition is sometimes reported. We would like to point out that the real solar panels are always encapsulated to protect the module from rainfall, dust and mechanical damage, so the air stability should not be a primary concern. Actually, tests done in the highly humid condition should be part of the accelerated aging tests.

In the next step approaching industrial standards (such as IEC 61215 and 61646 for crystal and thin film solar cells, respectively), damp heat condition like “85 °C and 85% relative humidity for 1000 h operational lifetime” should be implemented, as also suggested by Nazeeruddin et al.^17^. This can be done in association with the protocol of accelerated aging tests for perovskite solar cells. Real time tests of 2000 and 20,000 h take three months and two years to complete, respectively. It is infeasible and unacceptable to do a 2-year real time tests before publishing the results so the accelerated aging tests is the way to go.

To avoid the uncertainty caused by the encapsulation process, an easier way of conducting accelerate test is to test at 85 °C on a hot plate or in an oven under the dry nitrogen environment, with or without light soaking. In the meantime, the encapsulation technique is readily available from the OLED industry and enables more stringent and systematic tests. Following the industrial standards, the device should be eventually measured as a function of a series of higher temperatures, light intensity and humidity etc. The current goals are firstly to build the relationship between the accelerated aging lifetime and the real lifetime for PSCs and secondly to achieve 1000 h lifetime (80% retention of its initial efficiency) under 85 °C and 85% relative humidity. We believe the stability research is of vital importance to bring the perovskite technology to real applications.

## The “real” cost of PSCs

Levelized cost of energy (LCOE) is a good, but not yet perfect performance index that can reflect the cost competitiveness and potential attractiveness of the PV technology^18,19^. LCOE is defined as the net present value of the unit-cost of power (in US cents kWh^–1^) over the lifetime of a power generating asset, such as a solar plant, wherein the unit-cost of power equals to the total cost divided by total power output. The nice thing about LCOE is that it takes into account all the three key parameters in the PV golden triangle. If the module PCE increases, it leads to proportionally higher total power output that approximately cuts off the unit cost of power, i.e., lowers the LCOE in proportion. Alternatively, LCOE can be cut off by prolong the lifetime of the PV module. Here we show the dependence of estimated LCOE of perovskite PV on the device lifetime (Fig. 2b) which is also close to “inverse proportional”. Given the similar dependence of LCOE on PCE and lifetime, the community should therefore shift the focus on to the stability study given the fact that the efficiency is nearly saturated. To be competitive with the dominating c-Si PV (PCE of 21%), LCOE of perovskite PV has to hit 5.50 US cents kWh^−1^. Based on the LCOE calculation, we suggest that lifetime of 15 years is the threshold for perovskite PV (with a PCE of 19% and module size of at least 100 cm^2^), which is a long way to go from current status.

In conclusion, perovskite PV has demonstrated good processability and high efficiency compared to conventional PV and the stability issue seems to be the last technological barrier for its commercialization. Rigorous research efforts on material development and device engineering are demanded to achieve both high efficiency and longer lifetime, guided by on-going studies of the degradation mechanism in parallel. In the meantime, we urge the community to consider adopting standardized protocols to characterize perovskite solar modules and thus making fair comparisons between results, based on which the performance metrics could thus be contextualize towards future commercial applications.
